# The Application of Wireless Underground Sensor Networks to Monitor Seepage inside an Earth Dam

**DOI:** 10.3390/s23083795

**Published:** 2023-04-07

**Authors:** Min-Chih Liang, Hung-En Chen, Samkele S. Tfwala, Yu-Feng Lin, Su-Chin Chen

**Affiliations:** 1 Department of Soil and Water Conservation, National Chung Hsing University, 145 Xingda Road, Taichung 40227, Taiwan; d106042001@mail.nchu.edu.tw (M.-C.L.); hechen@email.nchu.edu.tw (H.-E.C.); 2 Department of Geography, Environmental Science and Planning, University of Eswatini, Kwaluseni M201, Eswatini; sstfwala@uniswa.sz; 3 Department of Civil and Construction Engineering, National Taiwan University of Science and Technology, No.43, Keelung Rd., Sec.4, Da’an Dist., Taipei City 106335, Taiwan; d11105001@mail.ntust.edu.tw; 4 Innovation and Development Center of Sustainable Agriculture (IDCSA), National Chung Hsing University, 145 Xingda Road, Taichung 40227, Taiwan

**Keywords:** seepage in earth dam, LoRa, multi-hop transmission, wireless transmission

## Abstract

Earth dams or embankments are susceptible to instability due to internal seepage, piping, and erosion, which can lead to catastrophic failure. Therefore, monitoring the seepage water level before the dam collapses is an important task for early warning of dam failure. Currently, there are hardly any monitoring methods that use wireless underground transmission to monitor the water content inside earth dams. Real-time monitoring of changes in the soil moisture content can more directly determine the water level of seepage. Wireless transmission of sensors buried underground requires signal transmission through the soil medium, which is more complex than traditional air transmission. Henceforth, this study establishes a wireless underground transmission sensor that overcomes the distance limitation of underground transmission through a hop network. A series of feasibility tests were conducted on the wireless underground transmission sensor, including peer-to-peer transmission tests, multi-hop underground transmission tests, power management tests, and soil moisture measurement tests. Finally, field seepage tests were conducted to apply wireless underground transmission sensors to monitor the internal seepage water level before an earth dam failure. The findings show that wireless underground transmission sensors can achieve the monitoring of seepage water levels inside earth dams. In addition, the results supersede those of a conventional water level gauge. This could be crucial in early warning systems during the era of climate change, which has caused unprecedented flooding events.

## 1. Introduction

Earth dams, also known as “embankment”, “earthfill”, or “rockfill” dams, have received significant attention due to their popularity in disaster phenomena [1]. The failure of earth dams can pose a significant risk to the environment and nearby communities, as they can release large amounts of contaminated water and sediment downstream. According to data from the International Commission on Large Dams (ICOLD) and the literature [2], there were over 1600 recorded dam failures worldwide between 1800 and 2010, of which a significant proportion were embankment dams. Several forms of embankment dam failure are reported, such as overtopping, foundation defects, piping, or structural failure of the materials. Seepage and internal erosion within an embankment dam are some of the leading causes of instability [3]. When the seepage of a dam exceeds the critical value, internal erosion or structural instability occurs, leading to collapse [4]. Therefore, detecting seepage paths is crucial for preventing potential earth dam collapses and establishing anti-seepage measures [5,6].

Most dam seepage monitoring methods can be divided into three categories: internal monitoring, environmental monitoring, and slope monitoring. Internal monitoring using geophysical methods has the advantages of being fast, non-destructive, and low-cost but is limited by technical operational characteristics and the need for extensive wiring. Methods in this category include electrical resistivity tomography (ERT), electromagnetic (EM) methods, seismic refraction tomography (SRT), ground-penetrating radar (GPR), and earthquake noise monitoring [4,5,7,8,9,10,11,12,13]. Internal monitoring can provide detailed information about the interior of the dam, but it still has drawbacks, such as a limited depth and the inability to conduct long-term monitoring. Environmental monitoring mainly focuses on the hydrological and geological environment around the dam, including upstream water level monitoring, rainfall monitoring, visual observation [14], and deformation measurement [15], which are long-term monitoring activities. Slope monitoring focuses on temperature monitoring on the surface of the dam, and commonly uses thermal imaging [16,17] and fiber-optic distributed temperature sensing (DTS) [18,19].

From the above literature, traditional wired transmission is commonly used for monitoring systems. Wired transmission equipment includes electrical and fiber-optic cables, which can provide a long-term power supply for the equipment. Moreover, it requires on-site cable infrastructure, which is difficult to maintain and has high deployment and construction costs. In addition, deploying a large amount of wiring for wired communication will lead to negative aesthetic effects [20,21]. Recently, several studies have shown that wireless sensor networks (WSNs) are more scalable, may be quickly deployed, reduce construction difficulty, increase system flexibility, and will even adapt to diverse application scenarios [22,23]. Since earthen dams are mostly located in remote and sparsely populated areas, it is difficult to obtain power equipment and carry out immediate maintenance; henceforth, using wireless transmission would be the preferred choice. 

The remaining sections of this article are organized as follows. Section 2 discusses related work. Section 3 provides detailed explanations of the materials used to develop the sensors, the transmission methods, and the evaluation of the transmission results. Section 4 details the methods applied for point-to-point transmission, hop-by-hop transmission, and power testing, as well as the transmission results. Section 5 provides a detailed introduction to the configuration of the dam, water level gauge, and sensors used in the seepage experiment, as well as the results of the seepage experiment. Section 6 discusses the experimental results from Section 4 and Section 5. Finally, Section 7 concludes the article.

## 2. Related Work

In recent years, the advancement of the Internet of Things (IoT) technology has made it possible for monitoring devices to collect data from different environments. The application areas include agricultural data collection [24,25,26], water quality monitoring [27], industrial control, flood prevention [27], smart water network management [28], environmental monitoring [22], and healthcare [29]. These IoT devices’ sensors, computing, and communication form wireless sensor networks (WSNs) [30]. A WSN is a network that can interconnect multiple sensor nodes wirelessly. Sensors are typically small and low-power devices, and are very varied, including environmental sensors, seismic sensors, low-sampling-rate magnetic sensors, thermal sensors, and visual sensors [22]. They can be widely deployed in various areas, making WSNs widely used in multiple fields. When using multi-hop communication, WSNs can consume less power than traditional single-hop communication and will effectively overcome long-distance transmission challenges [22,31]. Compared to ground wireless sensor networks, WUSN technology can provide better concealment, ease of deployment, real-time data, reliability, and coverage density [21]. It has been successfully applied in military tracking, healthcare, infrastructure monitoring, post-natural disaster human rescue, and smart agriculture [21,32,33]. The communication types under WUSN can be divided into three types: underground to underground (UG2UG), underground to aboveground (UG2AG), and aboveground to underground (AG2UG) [32,34,35]. The advancement of wireless communication technology has led to the development of various products, including LoRa, ZigBee, and NB-IoT [35,36,37,38], which are all part of low-power wide-area networks (LPWANs). These have the potential to provide a large number of devices over long distances with low power consumption, offering a potential solution for WUSN applications [39,40]. In wireless communication technology, LoRa (Long Range) has a remote communication capability that utilizes chirp spread spectrum (CSS) technology [41]. LoRa was explicitly developed for long-range and low-power IoT applications and supported license-free radio frequency bands. This technology can withstand high levels of interference and multi-path propagation, and is not affected by Doppler effects. This makes LoRa suitable for transmission in WUSN; however, the limiting conditions of LoRa transmission in soil still need to be analyzed [42]. Nonetheless, the above-mentioned environmental monitoring related to dam monitoring is mostly applied on the ground and less devoted to underground monitoring. This study aims to fill this gap by integrating environmental monitoring and internal monitoring. Moreover, wireless communication in dense materials such as soil or rock is more challenging than wireless communication through the air, as noted by Akyildiz and Stuntebeck [21]. Wan, Yang, Cui, and Sardar [42] conducted single-point wireless underground propagation testing on the soil surface and proposed some suggestions for applying LoRa-based wireless underground sensor networks (WUSNs) in soils. The concept of mesh multi-hop routing has been proposed to overcome the challenges associated with underground transmission. Ebi et al. [43] demonstrated the feasibility of data transmission for urban drainage systems through mesh multi-hop routing. Abrardo and Pozzebon [20] proposed a linear sensor based on multi-hop LoRa chain communication, and conducted feasibility testing in the underground excavation of a medieval aqueduct environment. 

Regarding earth dams, seepage and internal erosion are the main factors causing embankment instability. The lake water level before embankment failure, as well as the pore pressure or water content inside the embankment, can be used as the basis for early warning of embankment failure. If changes in the water level or water content inside the embankment can be monitored in real time by overcoming the bottleneck of wireless underground transmission, then the safety of downstream areas and property can be effectively ensured. This study aimed to develop a wireless underground transmission sensor module based on IoT technology, equipped with a soil moisture sensor. LoRa was chosen as the communication protocol due to its long-distance, low-power, low-cost, and license-free characteristics. After the sensor communication module was developed, peer-to-peer transmission tests, multi-hop underground transmission tests, power management tests, and soil moisture tests were conducted to propose a feasible configuration for underground transmission based on the experimental results. The wireless underground transmission sensor was then applied to monitor changes in soil moisture inside the embankment, and the measurement results were compared with those obtained from water level sensors. The findings from this study take a leap towards preventing large disasters related to earth dam failure. 

## 3. Proposal

This section introduces the internal and external design of wireless underground transmission sensors, the architecture of program coding, power management instructions, methods for evaluating sensor performance, and explanations of soil moisture measurement testing.

### 3.1. Sensor External and Internal Design

**Figure 1:**
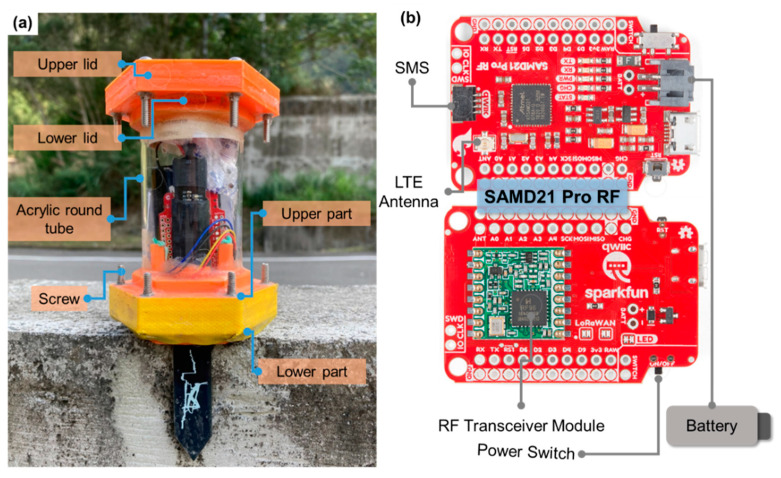
Wireless underground transmission sensor: (**a**) external configuration and finished product of the sensor: (**b**) internal configuration.

In this study, a wireless underground transmission sensor equipped with a soil moisture sensor was developed for monitoring changes in the soil moisture content inside an earth dam. In terms of the external design of the sensor (Figure 1a), the sensor is designed to be used in an underground environment composed of materials such as water, metal, organic matter, clay, and gravel, which have high moisture and corrosion resistance. Therefore, the outer casing material needs to have moisture-proof and corrosion-resistant functions. The composition of the outer casing uses PLA plastic and acrylic. The upper lid, upper part, lower lid, and lower part of the outer casing are printed using a 3D printer with PLA material. Acrylic is used as the circular tube in the center of the outer casing, which facilitates observation of the sensor’s condition. The external dimensions are 17.8 cm in length and 8.5 cm in width. For waterproofing, an O-ring anti-corrosion oil seal and epoxy resin AB glue isolation (requires 24 h of static placement) are used, and, finally, screws are used for locking. For the internal design (Figure 1b), a SAMD21 Pro RF development board, soil moisture sensor (SMS), LTE antenna 100 mm FPC U.FL (flexible, 3 dBi gain, <3 VSWR, and 50 Ω impedance), desiccant, and lithium-ion battery are included.

### 3.2. SAMD21 Pro RF (SAMD21)-Based Sensor

The sensor development board used in this project is manufactured by SparkFun Electronics and is called the SAMD21 Pro RF. It has the advantages of being reliable and low-cost (approximately USD 33.95 per unit, 2022 estimate). The board comes with an RF transceiver module that supports the LoRa communication protocol and operates in the LoRa 923 MHz frequency band. The board has 4 digital and 5 analog IO pins, each with a dedicated GND pin and 256 KB of flash memory. The antenna is connected using a u.FL antenna connector. It has a power switch and a JST connector (2 pin) for power supply from a lithium battery. It can also be powered using a micro-USB connector. The power switch in the off position also allows for charging the connected lithium battery. The edge of the circuit board has a Qwiic connector, which makes it very easy to add sensors and actuators without the need for soldering or a soldering iron. The SAMD21 development board allows users to use the C++ programming language to program it in the Arduino IDE-based operating system.

### 3.3. Power Management Testing

Good power management can increase the device operating time and reduce maintenance costs. Sensor power management can be divided into sleep mode and the power-saving mechanism. Sleep mode mainly puts the sensor and the internal components of the development board into sleep. The power-saving mechanism mainly saves power during the entire device operation process by planning the timing of entering sleep mode based on the sensor’s requirements. Generally, manufacturers of sensors and development boards will consider sleep mode functionality when developing the products, and sleep mode can be achieved by calling the corresponding code in the program. Sleep mode will put the LoRa chip, SAMD21 chip, and soil moisture sensor into sleep. Planning for the power-saving mechanism needs to be written in the program’s operating system, and it can design the duration of work per hour and the number of work times. By changing the transmission operation time, the time that the device can maintain in sleep mode per hour can be increased.

### 3.4. Programming Plan for Wireless Underground Transmission Sensors

**Figure 2:**
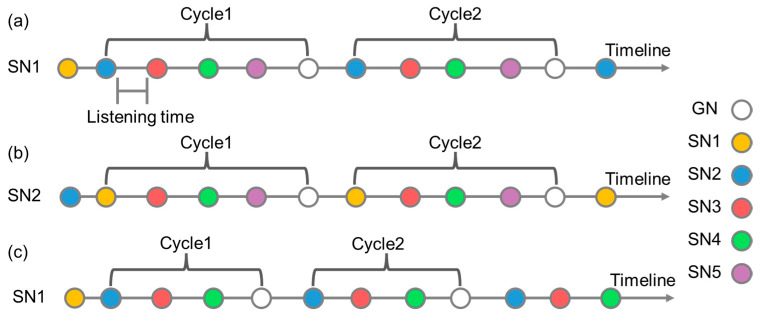
The process of forwarding message packets by the SN in order: (**a**) using SN1 as an example; (**b**) using SN2 as an example; (**c**) the process of forwarding message packets by the SN after dynamically adjusting and removing the failed SN5 (using SN1 as an example).

The firmware is programmed using the Arduino IDE, and the program design is divided into two parts: the Gateway Node (GN) and the Sensor Node (SN). The GN’s functions include receiving message packets sent by the SN, sending time information to the SN for calibration, and providing SN wake-up/sleep system decisions. The message packets received by the GN can directly present the data results through the computer. The SN is the bridge for sensor monitoring, sending monitoring data, monitoring power information, accessing other SNs, providing other SN message packet transmissions, dynamically adjusting the SN access order, and executing the wake-up/sleep system. The SN has an internal clock that can be updated through the time information provided by the GN and used as a trigger for SN wake-up/sleep system decision making. As shown in Figure 2a, taking SN1 as an example, the message packets are forwarded in the order of SN2→SN3→SN4→SN5→GN, and when the forwarding order is completed, one cycle is completed, with an interval of listening time after forwarding. As shown in Figure 2b, taking SN2 as an example, the order of message packets is changed to SN1→SN3→SN4→SN5→GN. When SN5 experiences a disruption event during monitoring (such as flooding, power failure, or entering sleep mode), the sensor can dynamically adjust the access order, remove unresponsive SNs, and reduce the forwarding time. As shown in Figure 2c, taking SN1 as an example, the sending order of SN1’s message packet is changed to SN2→SN3→SN4→GN. The reception time of the GN and SN is determined by generating a random value within a certain time range, and the probability of collision between data is reduced by using the randomly generated time.

**Figure 3:**
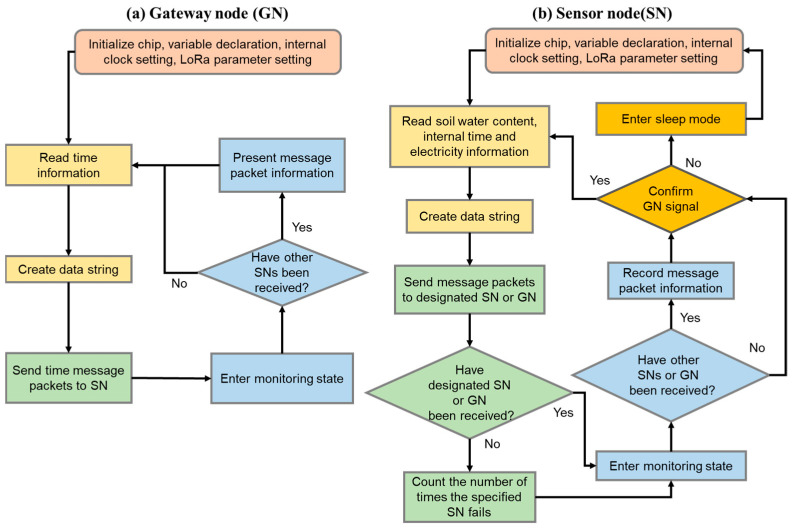
Wireless underground transmission sensor programming flowchart: (**a**) gateway node (GN) process; (**b**) sensor node (SN) process. First stage—pink; second stage—light yellow; third stage—green; fourth stage—blue; fifth stage—deep yellow.

The operation process of the GN can be divided into four stages (Figure 3a). The first stage is the system startup, which initializes the chip, declares variables, and sets the internal time and LoRa parameters (bandwidth: 125 kHz; coding rate: 4/5; spreading factor: 7). In the second stage, the internal time information is read and converted into a string. The time message packet is forwarded to the specified SN in the third stage. In the fourth stage, the transmission end becomes a receiving end and enters the listening state, waiting for about 7–9 s to determine if a reply is received from other SNs. When a reply from an SN is received, the message packet is immediately displayed on the computer. Regardless of whether a message packet is received in the fourth stage, the process will return to the second stage.

The operation process of the SN can be divided into five stages (Figure 3b). The first stage is the system startup to initialize the chip, declare variables, and set the internal time and LoRa parameters (bandwidth: 125 kHz; coding rate: 4/5; spreading factor: 7). In the second stage, it reads the soil moisture data, internal time information, and battery information, and converts the information into a string. In the third stage, the message packet is forwarded to a designated SN or GN. The purpose of forwarding to a designated SN is to provide a basis for dynamic adjustment and confirmation of the hop connection. When the forwarded SN fails to receive the message packet successfully, it will record one reception failure. The SN transmission target will be removed when the failure count exceeds 8 times. The purpose of forwarding to a GN is to pass the message packet to the computer for recording. In the fourth stage, it enters the listening state, and the transmitter becomes the receiver, waiting for about 5–7 s to determine whether there is a response from other SNs or GNs. When a time message packet from a GN is received, the time message will update the SN’s internal clock. When a message is received from the SN, the SN dynamic adjustment record will be recalculated. In the fifth stage, it will evaluate whether to enter sleep mode. After completing one round of message packet forwarding, it will judge whether a message has been received from a GN. The SN will enter sleep mode to save power when no GN message is received after more than 5 cycles.

### 3.5. Effectiveness Evaluation of Transmission Testing

The wireless underground transmission sensor developed in this study is mainly designed to overcome the challenges associated with underground transmission. Therefore, field tests were conducted to investigate the maximum transmission distance and transmission conditions of the sensor underground. During the transmission process, the received signal strength indication (RSSI) and packet loss ratio (PLR) of each node were observed, and the multi-hop ratio (MHR) was added for multi-hop transmission. Additionally, the RSSI has been used as a measurement to evaluate the signal quality in many wireless transmission studies [26,44]. The PLR represents the ratio of transmission failures to the total number of transmissions, calculated using the following formula:

(1) $$\mathrm{PLR}=\frac{T}{S}\times 100$$

where *T* represents the number of transmission errors, *S* represents the total number of transmissions, and PLR represents the packet loss ratio. The MHR represents the percentage of successful transmissions that experienced a multi-hop, calculated as the ratio of the number of hops to the total number of transmissions.

(2) $$\mathrm{MHR}=\frac{J}{S}\times 100$$

where *J* is the number of times that a hop occurred during transmission, *S* is the total number of transmissions, and MHR is the multi-hop ratio.

### 3.6. Soil Weight Water Content Law Test

The wireless underground transmission sensor developed in this study is equipped with an I2C soil moisture sensor (SMS), which uses the Inter-Integrated Circuit (I2C) protocol to transmit digital signals. The SMS converts the analogue signal to a digital signal through its internal firmware. The principle of the soil moisture sensor is to measure the dielectric constant in the soil, and the value of the soil moisture sensor is related to the soil properties. The soil moisture sensor available on the market uses standard sand to calibrate the relationship between the digital signal and the volume water content of the soil. However, the conventional density bottle experiment is limited because the local soil contains a large amount of gravel, making it difficult to carry out volume water content tests. Therefore, this study collected local soil and conducted indoor experiments to calibrate the relationship between the SMS digital signal and the weight water content of the soil. The test was carried out in five steps as follows: First, the local soil was placed in a pot with a diameter of 25 cm and a height of 10 cm (empty pot weight: 106 g), and the pot was placed in an oven (temperature set to 40 °C). After drying, the weight of the dry soil was recorded (5542 g). Secondly, water was added to the pot to cover the height of the soil, and the weight of the water was recorded (628 g). Thirdly, three self-made wireless underground transmission sensors were inserted into the soil, and the weight of the water and the soil moisture digital signal were recorded. Fourth, the pot was placed in the oven (temperature set to 40 °C), and after about 2 h, the pot was taken out, and the weight of the water and the soil moisture digital signal were recorded. The data acquisition time was set to 10 min. Step four was repeated until the soil moisture reached saturation. Finally, the relationship curve between the digital signal and the weight water content of the soil was analyzed and plotted.

## 4. Testing of Wireless Underground Transmission Sensors

A series of tests on wireless underground transmission sensors are introduced in this section, including peer-to-peer transmission tests, multi-hop underground transmission tests, power management tests, and soil moisture calibration tests. The test results are explained to demonstrate the effectiveness of developing wireless underground transmission sensors through these tests.

### 4.1. Peer-to-Peer Transmission Test

**Figure 4:**
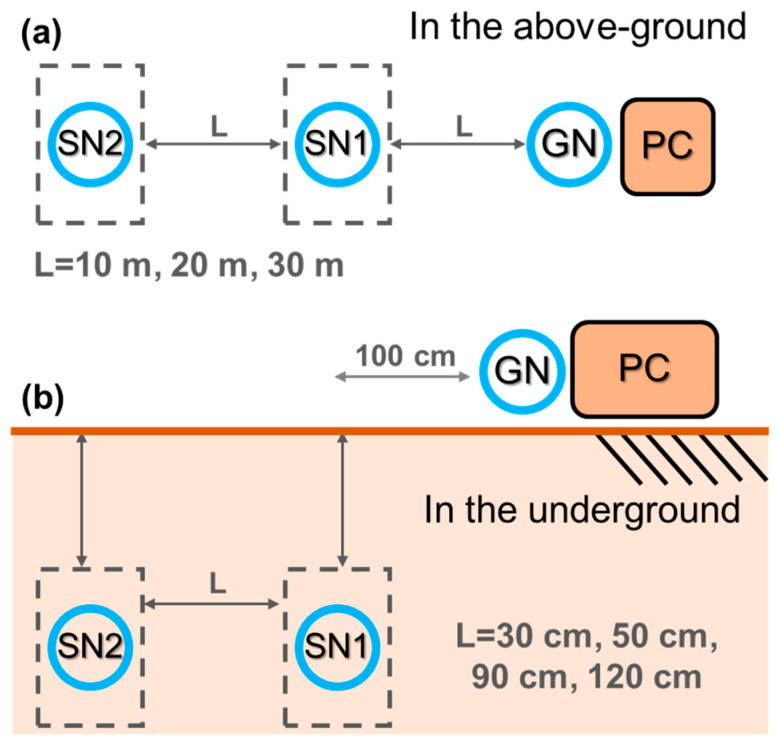
Peer-to-peer transmission test sensor configuration diagram for (**a**) ground transmission and (**b**) underground transmission.

Using peer-to-peer transmission tests to investigate the effects of different distances and depths on GN and SN transmission, the transmission mechanism was carried out in non-hopping mode, using two SNs and one GN, and the data acquisition time was set to 10 min to evaluate the RSSI and PLR measurement values of sensors sending signals to each other. PVC pipes protected the sensors to avoid soil contact that may cause damage. Peer-to-peer transmission tests were carried out in three different scenarios: aboveground to aboveground (AG2AG), aboveground to underground (AG2UG), and underground to underground (UG2UG). The AG2AG transmission tests were conducted at distances of 10 m, 20 m, and 30 m with the two SNs changing at equal distances (Figure 4a). The AG2UG and UG2UG transmissions considered four different distances and depths: 30 cm (depth of 30 cm), 50 cm (depth of 50 cm), 90 cm (depth of 90 cm), and 120 cm (depth of 90 cm), with the GN fixed aboveground at a distance of 100 cm from the SN1 plane (Figure 4b).

**Figure 5:**
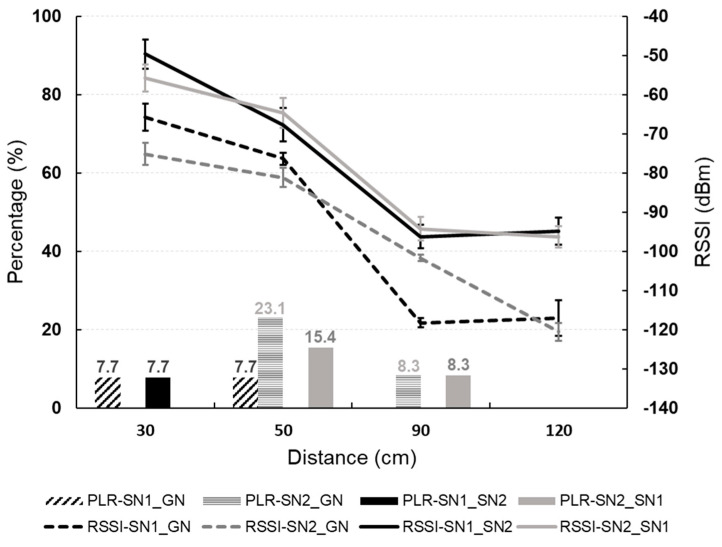
Peer-to-peer transmission test: the difference between the solid and dashed lines demonstrates signal attenuation caused by transmission through the different media.

The AG2AG transmission test results show that the average value of the RSSI was −76.33 (SDRSSI = 4), and the PLR was 8%. The values of the RSSI and PLR were very similar with changes in the distance, which indicates that the transmission of the sensors is minimally affected by the air medium. Referring to the transmission situation of the GN by SN1 and SN2, the AG2UG transmission test results (Figure 5) show that the RSSI decreased with increasing distance, and the variation in the PLR was not significant. The minimum RSSI was about −120. Based on the mutual transmission situation of SN1 and SN2, the UG2UG transmission test results show that the RSSI values within about 50 cm of the underground transmission were similar to those of the ground transmission, and the RSSI decreased significantly with increasing distance, with a minimum of about −95, suggesting that the soil medium significantly affects the transmission of the RSSI. Comparing the results of the AG2UG transmission and UG2UG transmission, the RSSI value of AG2UG was significantly lower than that of UG2UG, which indicates a significant loss effect as the transmission medium changes from air to soil. In summary, the underground transmission signal decreased with increasing distance, and there was a significant loss effect when the signal passed through different media (air and soil).

### 4.2. Multi-Hop Underground Transmission Test

**Figure 6:**
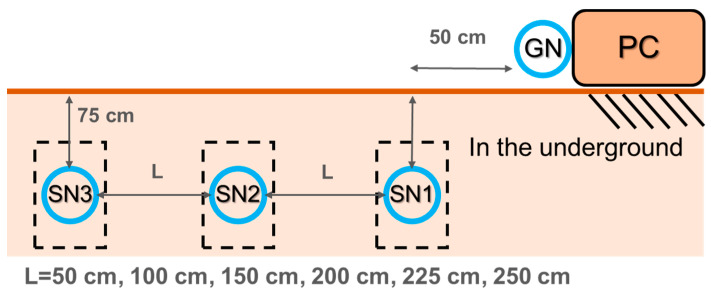
Experimental setup diagram of the multi-hop underground transmission test.

The multi-hop underground transmission experiment was conducted under the same depth but different horizontal distances to investigate the effect of signal hopping on the transmission of the GN and SN, and to measure the maximum distance of underground transmission. Three SNs and one GN were used in the experiment, with the SNs protected by PVC pipes and the data collected every 10 min. The SNs were placed at the same depth with only the distance between them varying evenly (Figure 6). All SNs were buried at a depth of 75 cm, with SN1 fixed at 50 cm from the GN, and six different horizontal distances were considered: 50 cm, 100 cm, 150 cm, 200 cm, 225 cm, and 250 cm. The experiment was designed for the GN to receive data, and the average RSSI, PLR, and MHR were estimated by receiving data from SN1 and SN2.

**Figure 7:**
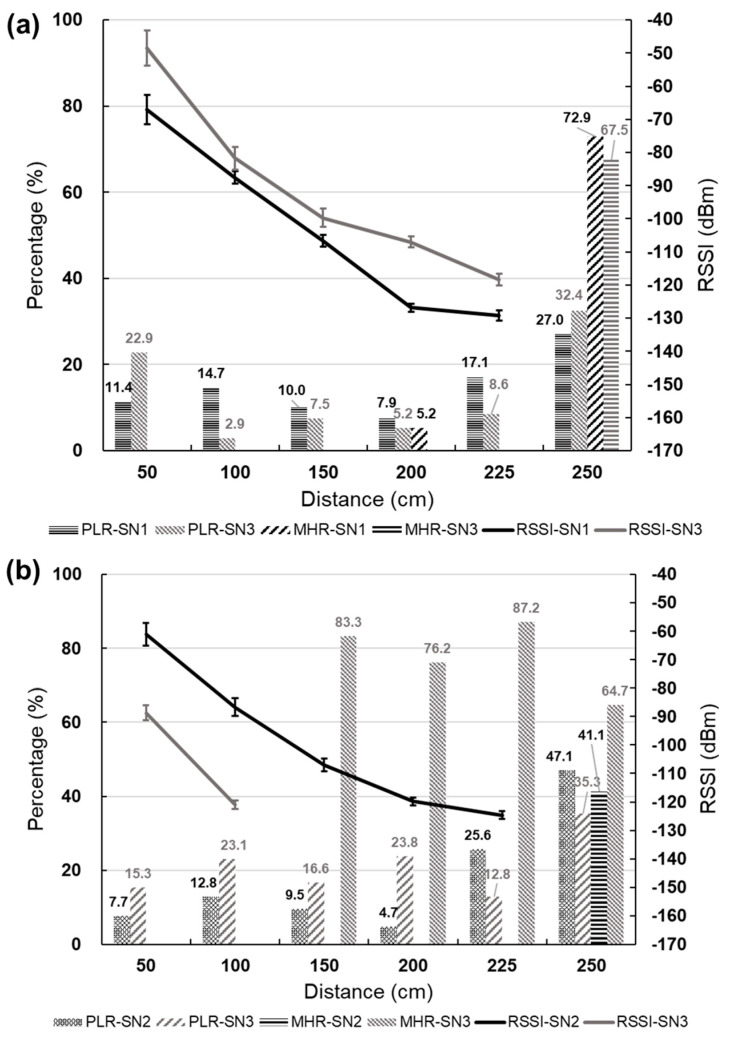
Multi-hop underground transmission test: (**a**) transmission status of SN2 to SN1 and SN3; (**b**) transmission status of SN1 to SN2 and SN3 (when RSSI information is not recorded after the multi-hop occurs).

The analysis of this test refers to the data results returned by SN2 and SN1. The analysis results of SN2 (Figure 7a) show that SN2 was located between SN1 and SN3, and the distance changes were the same. The transmission situation of SN2 to SN1 and SN3 can be observed. The RSSI gradually decreased with increasing distance. Since the distance changes were equidistant, the RSSIs of SN1 and SN3 showed the same trend. When the test distance reached 250 cm, the PLR of SN1 and SN3 increased significantly. SN1 and SN3 needed to communicate with SN2 through the hop point of the GN, indicating that the maximum transmission distance of peer-to-peer transmission converted to multi-hop transmission was approximately 225 cm, and that multi-hop transmission would increase the PLR. The analysis results of SN1 (Figure 7b) show the transmission situation of SN1 to SN2 and SN3. The RSSI gradually decreased with increasing distance. When the horizontal distance reached 150 cm, the distance between SN1 and SN3 was 300 cm, and SN1 needed to use SN2 and the GN as hop points to transmit to SN3. During the process of increasing the horizontal distance from 150 cm to 250 cm, SN3 had a very high MHR of about 80%, and the PLR changed slightly, indicating that the transmission behavior was mostly multi-hop transmission. When the horizontal distance was 250 cm, hop points occurred between SN1 and SN2, and SN2 needed to communicate with SN1 through the GN. The horizontal distance between SN1 and SN3 increased to 500 cm, causing multiple transmission failures during the transmission process. Therefore, the PLR of SN2 and SN3 increased significantly, and the MHR decreased. The test results show that the distance of 250 cm significantly impacts underground transmission.

### 4.3. Power Management Test

The wireless underground transmission sensor uses a lithium-ion battery to store power, with an output voltage of 3.7 V and a rated capacity of approximately 5200 mAh. As the battery’s lifespan decreases with usage, and it is impossible to confirm whether it is fully charged after charging, the estimated battery capacity for the sensor operation time is only 80% of the rated capacity (4160 mAh). The sensor’s power consumption is estimated using a combination of sleep mode and the sleep mechanism. The wireless underground transmission sensor’s average operating current is measured using a three-meter (3 m) meter, returning approximately 22.5 mA, which can be reduced to 0.4 mA during sleep mode. The sleep mechanism of the sensor is set to sleep for 1 h each time. After waking up, it operates for 7 min to search for surrounding sensors, send and receive messages, and confirm the GN signal source. If there is no GN signal source, it will enter 1 h of sleep. When the sensor operates according to the sleep mechanism and has no GN signal source, it can operate for approximately 64 days. When the sensor works continuously without sleep, it can last for approximately 7 days.

### 4.4. Soil Moisture Calibration Test

**Figure 8:**
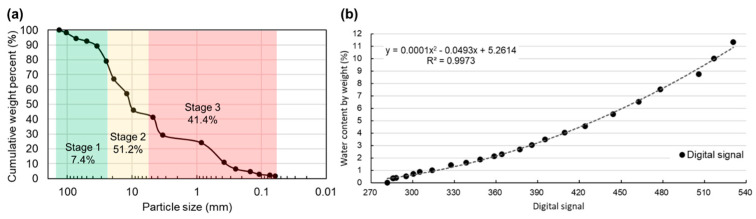
(**a**) Results of on-site soil sieving analysis. (**b**) Relationship between digital signal and weight-based soil moisture content graph.

A calibration test was conducted for soil moisture sensors using field soil, and the relationship curve between the digital signals and soil weight moisture content was plotted. Firstly, a three-stage sieving analysis test was performed on the field soil. The test results (Figure 8a) showed that in the first stage, any gravel with a length greater than 10 cm was taken, and its cumulative weight percentage was about 7.4%. In the second stage, a sieve mesh size range of 50 to 9.525 mm was used, and the cumulative weight percentage was about 51.2%. In the third stage, a standard sieve mesh size analysis was performed indoors, ranging from #4 (4.76 mm) to #200 (0.074 mm), and its cumulative weight percentage was about 41.4%. To avoid the non-uniformity of large and fine particles, the soil in the third stage was used to calibrate the soil moisture sensor. The soil at the test site had a water content of 3.8% and a dry unit weight of 22.56 (kN/m^3^). According to the ASTM classification, the soil falls under coarse-grained soil with gravel. Three sensors, SN1, SN2, and SN3, were used for the calibration tests, and the data acquisition time was set to 10 min. The calibration test results of the soil moisture sensor (Figure 8b) showed that the digital signal was in the range of about 520~240 (with an average standard deviation of 3.08) from full water to no water, and the soil weight moisture content was in the range of 12%~0% from full water to no water. According to the trend line, the digital signal can be converted into the soil weight moisture content through the regression formula in Figure 8b.

## 5. Seepage Test of Earth Dam

Field-scale seepage tests were conducted on the earth dam to test the actual deployment and performance of the wireless underground transmission sensors. The sensors monitored changes in the water content within the dam, and the results were compared with those obtained from monitoring wells. The following describes the layout of the field test and the results of the sensor measurements.

### 5.1. Configuration of Seepage Test

**Figure 9:**
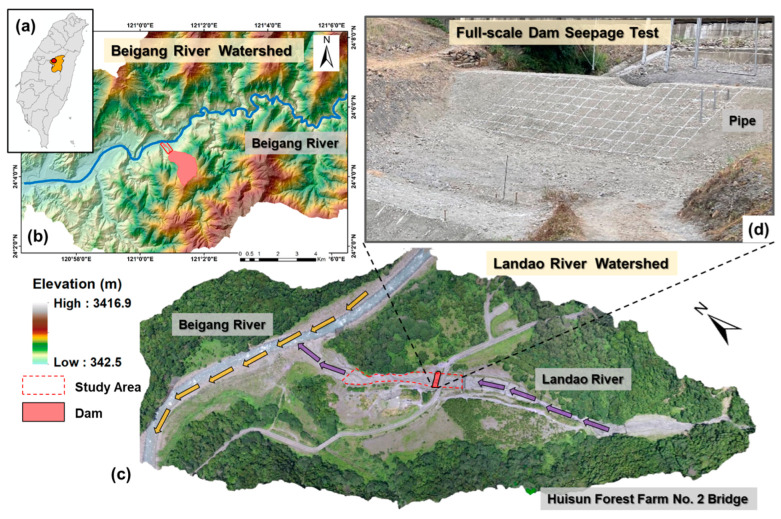
Study area: (**a**) Guoxing Township, Nantou County; (**b**) Beigang River watershed; (**c**) Lanjiang River watershed; (**d**) deposition condition of on-site earth dam.

**Figure 10:**
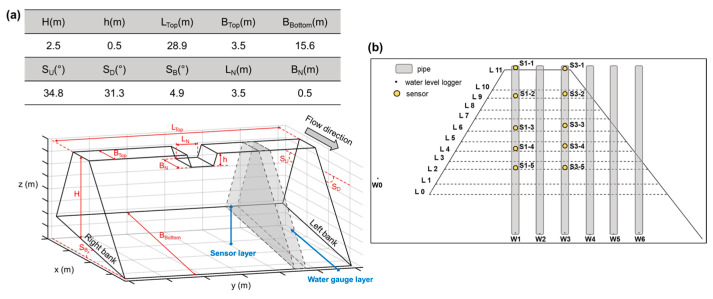
Seepage test of the earth dam: (**a**) dimensions of the earth dam and (**b**) location of sensors and water level loggers.

The study area was in the downstream section of the Landao River, a tributary of the Beigang River in Ren’ai Township, Nantou County (Figure 9a–c). The test section was approximately 280 m long, with an average channel width of 28 m and a slope of roughly 6.3°. During normal conditions, the channel was dry, and the outlet of a sediment outlet structure located upstream was used for the test. The dam was constructed by mechanically stacking soil, forming a closed dam (Figure 9d). The dimensions of the dam are shown in Figure 10a, where H represents the dam elevation, h represents the breach height, and other dimensions are indicated as per the image description. The soil was stacked in layers of approximately 25 cm, with a total of 11 layers. The breach was located on the 10th layer, and the 11th layer consisted of two wing dams, each approximately 50 cm high, on either side of the breach. The height of each layer and the coordinates of the sensors were recorded using a total station. A pressure-type water level logger (HOBO Water Level Logger U20-001-01) was also installed to monitor the water level. A PVC pipe was installed on the left bank of the dam to protect the water level logger. The sensors were arranged in a straight line inside the dam (Figure 10b), forming an independent transmission chain. Each transmission chain had a GN for data reception. One set of SN and GN transmission chain was placed on top of the dam (representing air medium). Two sets of transmission chains were buried inside the dam, with one set consisting of five SNs and one GN. The sensor and receiver numbers of the transmission chain were S1-1, S1-2, S1-3, S1-4, and S1-5 for the first set, and S3-1, S3-2, S3-3, S3-4, and S3-5 for the second set. Water level meters numbered W1, W2, W3, W4, W5, and W6 were used as a reference for the soil water content data. In addition, water level meter W0 recorded changes in the water level of the reservoir. The first set of transmission chains corresponded to water pipe W1, and the second set corresponded to water pipe W3. The sensors were placed starting 50 cm from the bottom of the dam, with S1-5, S1-4, and S1-3 placed at 50 cm intervals, and S1-1 and S1-2 placed at 75 cm intervals. The arrangement of the two sets of transmission chains was the same. To prevent damage of the instruments during the breach period, the instruments were buried in a location that would not be affected by the breach. They were placed at 1.55 m from the water pipe in the direction of the breach, with 1.3 m between the water pipes.

### 5.2. Analysis of Internal Water Content of Dam

**Figure 11:**
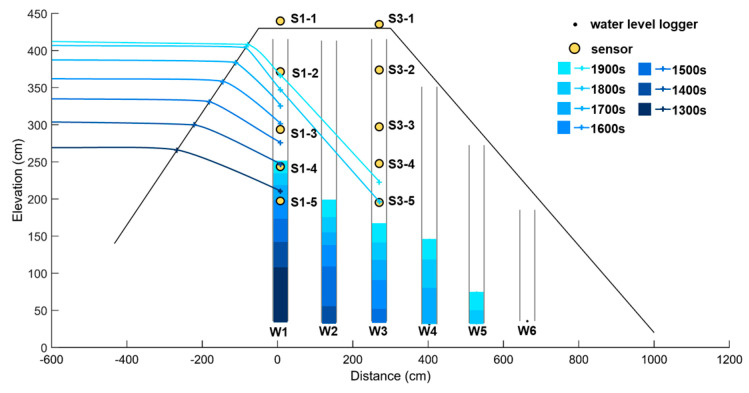
Analysis of seepage using water level data from the water gauge and monitoring data from the sensors in the earth dam seepage test.

**Figure 12:**
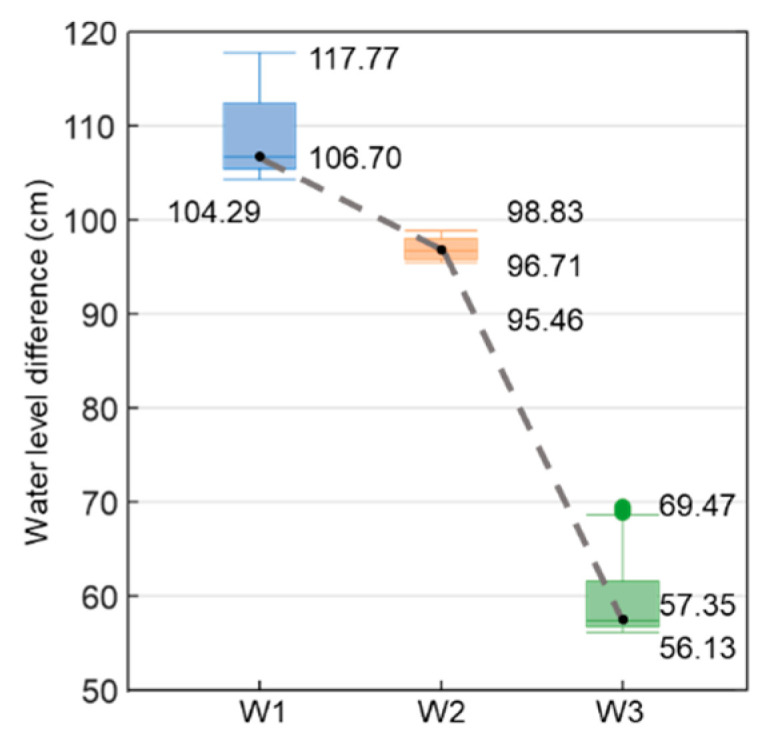
Box plot of water level differences between water level meters and sensors.

The experimental results compare and analyze the data obtained from the water level meters and sensors. The analysis results (Figure 11) show that the data were taken at 1300 s, 1400 s, 1500 s, 1600 s, 1700 s, 1800 s, and 1900 s, with the color depth becoming lighter as time elapsed. The time and location of the water level monitored by the sensors were interpolated, and the interpolated water levels were connected. In the first transmission chain, only the sensor of S1-1 did not detect any change in moisture. At 1300 s, the water level measured by the W1 water level meter and that monitored by the sensor were 70.20 cm and 175.19 cm, respectively, with a water level difference of 104.99 cm. The water level of the W1 water level meter did not reach the highest water level of the sensor after the experiment. In the second transmission chain, the sensors that detected soil moisture were S3-5 and S3-4. At 1800 s, the water level measured by the W3 water level meter and that monitored by the sensor were 103.30 cm and 160.61 cm, respectively, with a water level difference of 57.31 cm. The water level of the W3 water level meter also did not reach the location of the sensor after the experiment. The first and second transmission chains were interpolated, and the water level of the sensor in W2 was estimated, which was not reached by the water level after the experiment. This suggests a water level difference between the water level meter and the sensor, and that the water level meter cannot truly represent the seepage water level within the dam. According to the box plot showing the water level difference (Figure 12), the median water level difference between the sensor and the water level meter was 106.70 cm for the W1 water level meter, 96.71 cm for the W2 water level meter, and 57.35 cm for the W3 water level meter. The seepage test caused the dam to fail within about 30 min, so the observation time of the seepage changes was very short. However, the water level in the water well needs time to seep into the wall of the pipe, making it difficult to react as quick as the sensor. The water level difference between the sensor and the water level meter will decrease to the downstream water level meter, and the difference between the two will gradually narrow.

## 6. Discussion

A series of tests were carried out to investigate the feasibility of multi-hop transmission using the wireless underground transmission sensor. These tests included peer-to-peer transmission, multi-hop transmission, power management testing, and soil moisture testing. The sensor distribution location was not too dispersed to achieve more accurate monitoring of seepage water levels in the dam, so the LoRa parameters were set to the default medium-distance setting in the program. Although we did not optimize the LoRa parameters for different soil compositions and moisture content changes, they were sufficient to achieve the monitoring aims.

The peer-to-peer transmission test mainly investigated the influence of different environments (air and soil) and distance variations on transmission, and the evaluation was based on the measurement values of the RSSI and PLR. The AG2AG test results showed that the transmission RSSI and PLR of the sensor on the ground at a medium distance (60 m) were not affected by distance variations. Although this test did not investigate the transmission limit distance of peer-to-peer transmission in the air, previous research has shown that wireless transmission in the air can be achieved at distances of approximately 2 km [45], 8 km [46], and 22 km [47] through adjustments of different LoRa parameters and antenna heights, as well as application area environments. Adjustments to LoRa parameters (output power and spreading factor) will affect the receiver sensitivity, causing the RSSI to vary, and trade-offs between the distance and transmission data volume need to be considered before adjusting parameters [43,48]. These results show that LoRa has great application flexibility. The comparison between AG2AG transmission and UG2UG transmission showed that the RSSI value decreased with distance underground, indicating significant attenuation of the transmission RSSI value in the soil medium. The comparison between AG2UG transmission and UG2UG transmission showed that when the transmission signal medium changed from air to soil, the RSSI attenuation was significantly affected by the soil medium. The presence of plant roots and small amounts of gravel on the ground led to greater signal attenuation. These findings are similar to the test results of Wan, Yang, Cui, and Sardar [42].

The multi-hop transmission test aimed to investigate the transmission of sensors under the same depth and different horizontal distance conditions. The maximum transmission distance of the sensor, which changed from peer-to-peer transmission to multi-hop transmission, was about 225 cm, and the PLR showed a slightly upward trend. Similar case studies on underground transmission suggest that the transmission distance is affected by changes in the soil properties and water content [35,42]. According to Hardie and Hoyle [35], UG2UG transmission can reach up to 20 m. In addition, other scholars have suggested that reliable transmission can only be carried out within 7.5 m [34]. In this study’s seepage test of the earth dam, the main monitoring object was the seepage water level inside the dam. Considering that the sensor’s volume may affect the seepage result, a soft antenna minimizes the sensor’s volume. During the storage process, the sensor is placed circularly, which may lead to a shorter transmission distance. In the future, the antenna type may be adjusted according to the monitoring object. Based on the transmission results of SN1, when the distance between SN1 and SN2 reached 250 cm, the actual distance between SN1 and SN3 was 500 cm, and there was a significant upward trend in the PLR. Therefore, it was proved that the maximum distance that can maintain the PLR without being affected under the hop transmission is 450 cm. In addition, it was observed during data analysis that there is a waiting time for the response after the sensor sends data. The response time when the sending is successful is about 0.2 s, and 2 s~4 s when unsuccessful. Therefore, when the transmission distance of the sensor exceeds the limit, it is easy to increase the response time and affect the PLR. It is therefore recommended that in establishing the transmission chain of underground transmission in the future, the sensor configuration should be within the effective distance.

Wireless underground transmission sensor power management is achieved by using sleep mode and a sleep mechanism to save power. The sleep mechanism is set to 1 h of sleep time and 7 min of operation time. During the field test, sleep mode was canceled using the remote control. The total time spent on the construction of the embankment, installation of sensors, and measurement operations during the seepage test was 9 days. The sensor can operate normally during the seepage test by coordinating operating and sleep times. Ten wireless underground transmission sensors were used in the field test, which all successfully awakened during the test. The sleep mechanism used in this test was fixed-time-based (sleep–awake–sleep–awake), although the awakening was occasionally unnecessary. We propose to change the sleep mechanism such that the periods without awakening have a longer sleep time, and to adjust the battery capacity according to the monitoring items to achieve a longer battery life.

The soil moisture content test results showed a difference in the water level between the water level meter and the sensor. In addition, the water level difference between the sensor and the water level meter decreased downstream, proving there was a time delay in the reaction of soil water flow inside the embankment after entering the water pipe (groundwater well) to generate a seepage phenomenon. After the entire seepage test, the seepage water level monitored by the sensor was higher than that monitored by the water level meter, and the water level monitored by the water level meter did not reach the actual seepage height, indicating that the water level meter could not truly represent the seepage water level inside the embankment. Although the sensor transmission mechanism may cause the monitored soil moisture content to be non-real-time measurement results, the effect of expressing the actual seepage phenomenon of the embankment is better than the monitoring results of the water level meter.

Considering the limitations of the research method discussed above, when sensors are applied to earthen dams, on-site drilling is still required using machinery. As the transmission of the sensor is wireless, the size of the drilling hole only needs to be slightly larger than that of the sensor. In terms of power management, increasing the power capacity or changing the sleep mode can increase the lifespan of the device. However, in the case of this study, increasing the power capacity requires a larger sensor casing or expensive batteries, which would have a greater impact on seepage within the earthen dam. Optimizing the sleep mode has become an important task, but in the design of this study, it means increasing the sleep cycle, which can affect the timing of the sensor operation as the cycle increases.

## 7. Conclusions

This study developed a wireless underground transmission sensor equipped with a soil moisture sensor to monitor the seepage water level changes inside a full-scale dam body. Feasibility tests were conducted on the wireless underground transmission sensor, including peer-to-peer transmission tests, multi-hop underground transmission tests, and power management tests, followed by field seepage tests of the dam.

The peer-to-peer transmission test proved that the sensor signal did not attenuate when transmitted through the air over 60 m, and the effect of the RSSI and PLR in the air was smaller. The signal strength in the soil medium was greater than that in the air medium, and the signal decreased with distance. When the signal was transmitted, the conversion of different media resulted in a greater signal strength loss than that of a single medium. The multi-hop transmission test results proved that the maximum transmission distance of the wireless underground transmission sensor from peer-to-peer transmission to multi-hop transmission was about 225 cm, and that the transmission PLR would be significantly affected when the multi-hop transmission distance exceeds 250 cm.

The test results from the field seepage tests on the dam, using sensors and water level meters, showed that the water flow inside the dam’s soil would cause a delay in the seepage phenomenon after entering the water pipe (groundwater well). The wireless underground transmission sensor could monitor the seepage water level position faster. Due to the rapid changes in the dam’s breach water level, the water level gauge could not keep up with the seepage water level position. Furthermore, the water level difference between the sensor and the water level gauge decreased downstream. Therefore, this study proved that the wireless underground transmission sensor was better than the water level gauge in monitoring the actual seepage phenomenon in the earth dam, which could play a crucial role in future early warning systems. 

As part of our future work, we plan to conduct multi-hop transmission tests on different soil properties and environments, and propose parameter optimization adjustments for corresponding environments. We will propose better solutions for power management to improve the operational lifespan of sensors. Additionally, we will expand the monitored items of sensors, such as inclinometer sensors, pressure sensors, and vibration sensors, and construct a more comprehensive earth dam monitoring system.

## Data Availability

The data presented in this study are available on request from the corresponding author.
